# ‘What Does the Anemonefish Say?’: Investigating *Amphiprion percula* 's Acoustic Behaviour

**DOI:** 10.1002/ece3.72479

**Published:** 2025-11-14

**Authors:** Lucia Yllan, Theresa Rueger

**Affiliations:** ^1^ School of Natural and Environmental Science Newcastle University Newcastle upon Tyne UK

**Keywords:** anemonefish, animal behaviour, bioacoustics, social behaviour, social context

## Abstract

Acoustic communication plays a critical role in mediating social interactions, coordinating behaviours and maintaining group cohesion in many animals. While fish are known to produce a diverse range of sounds, most studies have been conducted in laboratory settings, limiting our understanding of how vocalisations function in natural social and ecological contexts. In this study, we provide a comprehensive examination of vocal behaviour in social groups of orange anemonefish ( *Amphiprion percula* ), an anemonefish species with strict size‐based hierarchies. Using underwater video and acoustic recordings from nine wild groups in Kimbe Bay, Papua New Guinea, we quantified acoustic features of individual vocalisations and linked them to behavioural contexts. We also examined the effect of body size and social status on vocalisation's acoustic features. Our findings demonstrate that *A. percula* 's vocalisations differ between behavioural contexts, highlighting the role of acoustic signals in signalling submission, regulating conflict, and maintaining group hierarchy. Behavioural context emerged as the strongest predictor of vocal variation, with body size and social rank providing additional variation. This study underscores the importance of field‐based investigations to capture the ecological and social complexity of acoustic communication. These findings establish a baseline for future functional studies, including playback experiments, and provide critical insight into the adaptive significance of sound in social fish communities.

## Graphical abstract

This study investigates the vocal behaviour of wild *Amphiprion percula* in Papua New Guinea, revealing that distinct acoustic signals correspond to specific social interactions and are influenced by behaviour and rank. The findings underscore the importance of in situ research for understanding fish communication and highlight potential disruptions from anthropogenic noise.

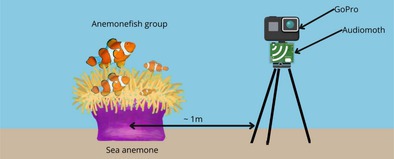

## 1 Introduction

With the evolution of sensory systems capable of detecting acoustic stimuli, many organisms also developed the ability to produce acoustic signals through diverse mechanisms. These signals are not emitted in isolation but are used in specific behavioural contexts to convey information (Margoliash and Hale 2008); for example, to attract mates, coordinate foraging or warn conspecifics of predators (Benoit‐Bird and Au 2009; Ibara et al. 1983; Zuberbühler 2009). Because of this, animal vocalisations provide valuable insights into social dynamics, ecological interactions, and life‐history processes. Bioacoustics has therefore become a powerful, non‐invasive tool for studying behaviour across taxa and for guiding conservation decisions (Teixeira et al. 2019). Crucially, however, to fully understand the function of acoustic cues, it is essential to observe and record animals in situ, where sounds are produced and perceived within the social and ecological contexts that shape their meaning.

Sound travels far more efficiently through water than light, and many marine animals have evolved to exploit this property for communication, navigation or foraging (Ladich and Winkler 2017). Cetaceans, for example, exhibit highly sophisticated acoustic communication, using complex vocalisations to coordinate social interactions and navigate their surroundings (Benoit‐Bird and Au 2009; Parks et al. 2014). Fish, despite lacking vocal cords or air‐filled resonating structures typical of many vertebrates, have independently evolved a diversity of sound‐producing mechanisms, including vibrations of the swim bladder and stridulatory structures such as teeth, fin rays or vertebrae (Fine and Parmentier 2015; Ladich and Fine 2006; Parmentier and Lecchini 2022). Over 800 fish species are known to produce sounds or vocalisations that serve a wide range of ecological functions, including predator alarms, courtship, territorial defence, group cohesion and predator deterrence (Bass et al. 1994; Ladich 1997, 2014, 2022). For instance, male *Porichthys* species generate continuous humming sounds during courtship to attract females to their nests (Ibara et al. 1983) and acoustic signals are also commonly used in agonistic interactions to reduce the risk of physical conflict or prevent escalations (Colleye and Parmentier 2012). Beyond these roles, sound may play a critical part in maintaining social structure and group cohesion among fish.

Despite decades of research on fish acoustic communication (Ladich and Winkler 2017; Bass and Ladich 2008; Amorim 2006) most studies have been conducted under laboratory conditions. While lab‐based experiments have provided foundational knowledge on hearing thresholds, sound production mechanisms and behavioural responses, they cannot capture the complex ecological and social contexts in which these vocalisations naturally occur (Calisi and Bentley 2009; Campbell et al. 2009). Laboratory settings may distort sounds through reflections off tank walls (Duncan et al. 2016), altering dominant frequencies and sound durations compared to recordings in open water (Akamatsu et al. 2002; Banse et al. 2023), and can constrain animals' interactions with their environment, thereby modifying natural social dynamics (Campbell et al. 2009). As a result, there is a critical gap in understanding how vocalisations function within undisturbed social groups, where factors such as hierarchy, habitat structure and environmental noise influence both sound production and perception. Observational studies conducted in the wild provide a more accurate view of the intricate interactions between individuals, their social structures, and their environment, capturing the complexity of these relationships in natural contexts (Archard and Braithwaite 2010; Turko et al. 2023). Therefore, in situ investigations linking acoustic signals with associated behaviours, social interactions and ecological context are essential for understanding the adaptive function of acoustic communication in fish.

Anemonefish (*Amphiprion* spp.) are an ideal model for studying field‐based acoustic communication due to their strict size‐based social hierarchies, group living within host sea anemones, and reliance on vocalisations for social interactions. Their obligate mutualistic relationship with sea anemones renders them highly site‐attached (Fautin 1991; Moyer and Nakazono 1978), allowing repeated observations and enabling researchers to study vocalisations within specific social and ecological contexts. Anemonefish groups exhibit a clear size‐based hierarchy, with non‐related individuals queuing for breeding positions (Buston 2004; Buston et al. 2007; Wong et al. 2007). The largest individual is the dominant female, followed by the sub‐dominant male that forms the breeding pair, which cohabits with non‐reproductive subordinates (Buston and Cant 2006). Acoustic cues are thought to be crucial for maintaining this hierarchy, signalling dominance and submission, and allowing individuals to be distinguished within the group (Rueger et al. 2021). Anemonefish can detect sounds between 75 and 1800 Hz (Parmentier et al. 2009) and produce vocalisations using their jaw teeth and rib‐cage vibrations, resulting in body size‐related variation in their acoustic signals (Colleye and Parmentier 2012; Colleye et al. 2009). Their vocalisations consist of short‐duration ‘pulses’ or ‘pops’ arranged into trains of consecutive sounds (Colleye and Parmentier 2012; Parmentier et al. 2007). These sounds are typically classified into two categories: aggressive vocalisations, often accompanied by threat postures and charge‐chases, and submissive vocalisations, usually paired with body shakes (Parmentier and Lecchini 2022). Previous studies have consistently found that body size correlates with acoustic features, with smaller individuals producing higher‐frequency shorter pulses and larger individuals producing lower‐frequency longer pulses (Colleye and Parmentier 2012; Colleye et al. 2009, 2011). However, these studies often did not explicitly account for social rank, which in size‐based hierarchies like those of anemonefish is tightly linked to body size. Because vocalisations may serve not only as indicators of body size but also as signals for maintaining social status and hierarchy, neglecting social rank limits our understanding of their functional significance within natural social groups.

Here, we provide one of the first comprehensive in situ studies of vocalisations and associated behaviours in wild anemonefish ( *Amphiprion percula* ) groups. By combining underwater video with acoustic recordings, we quantify the acoustic parameters of individual vocalisations and relate them to specific behaviours and social rank, establishing a baseline for future functional and playback experiments. This study addresses the gap in field‐based research and provides critical insight into the role of acoustic communication in maintaining social structure in natural marine environments. In this study, we aim to expand the knowledge on the role of sound in wild anemonefish social groups and behaviour, using groups of *A. percula* . We hypothesise that: (i) Vocalisations are used in a range of behavioural contexts and that there are distinct differences between vocalisations related to certain behaviours; we predict that behaviours may differ in the number of pulses of the vocalisations that accompany them (Amorim et al. 2015); (ii) The frequency and duration of the vocalisations are determined by individuals' body size (Colleye et al. 2009); (iii) Social status has an effect on vocalisation's acoustic features which is independent from body size; we predict that individuals might exhibit differences in their vocalisation to reflect their status within the group (Yllan et al. 2024).

## 2 Methods

### 2.1 Study Site

The study was conducted from February to April 2023 in Kimbe Bay, Papua New Guinea (5°30′ S, 150°05′ E). A total of 142 vocalisations (594 individual pulses) and associated behaviours were recorded in situ (8 AM–5 PM) from nine wild *A. percula* groups of two (*N =* 1), three (*N =* 3), four (*N =* 3) and five (*N =* 2) individuals, associated with the anemone *Radianthus magnifica*. The groups were distributed across seven different inner reefs in Kimbe Bay.

### 2.2 Behavioural and Acoustic Data Collection

**FIGURE 1:**
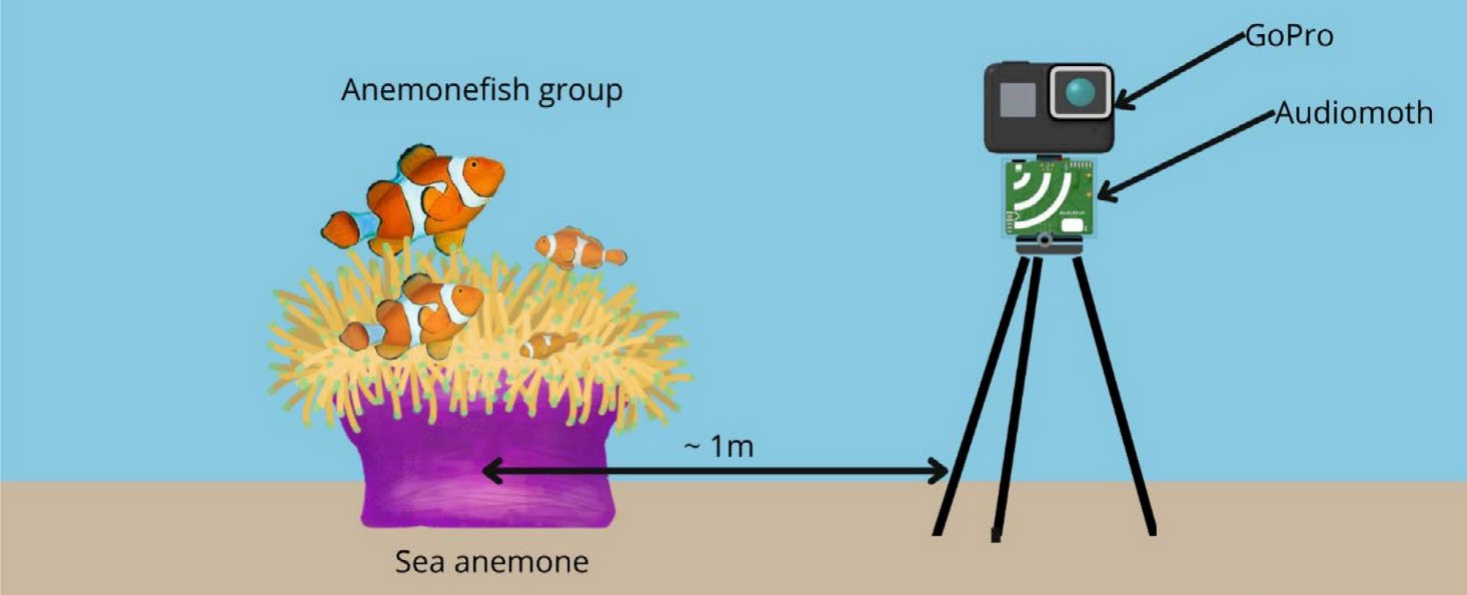
Experimental setup showing GoPro Hero 9 action camera and Audiomoth v1.7.1 acoustic logger mounted on a stationary tripod and positioned within 1 m of an anemonefish group and its host anemone for simultaneous video and audio recordings.

**FIGURE 2:**
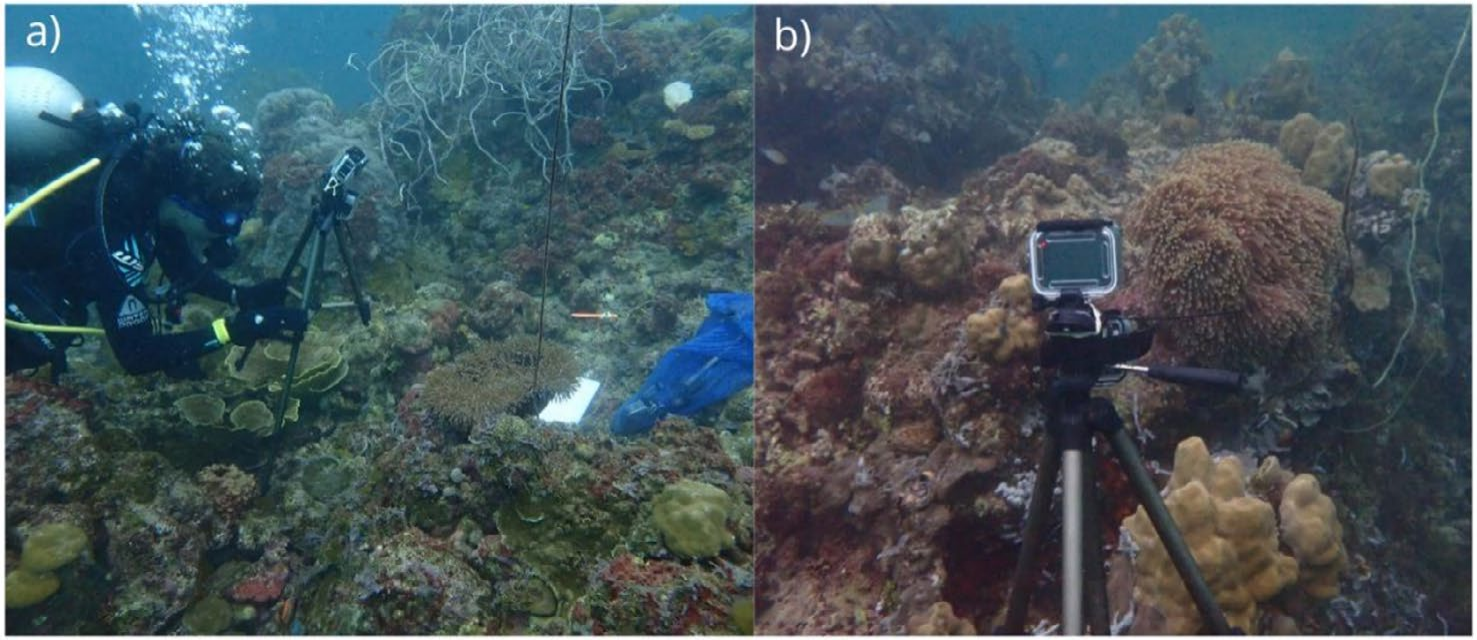
Images of experimental setup on the field. Photo (a) shows the diver deploying the GoPro camera and Audiomoth attached to the tripod within a meter of the sea anemone. Photo (b) depicts how the camera was situated to ensure the entirely of the sea anemone was within frame.

Each anemonefish group was recorded for 20 min using GoPro Hero 9 action cameras and AudioMoths version 1.7.1 (acoustic logger, Hill et al. 2018) that were placed by SCUBA divers in front of groups on stationary tripods (Figures 1 and 2) set within a meter of the anemone to obtain video and audio data, respectively. After set‐up, SCUBA divers would leave the area to ensure undisturbed recordings.

**FIGURE 3:**
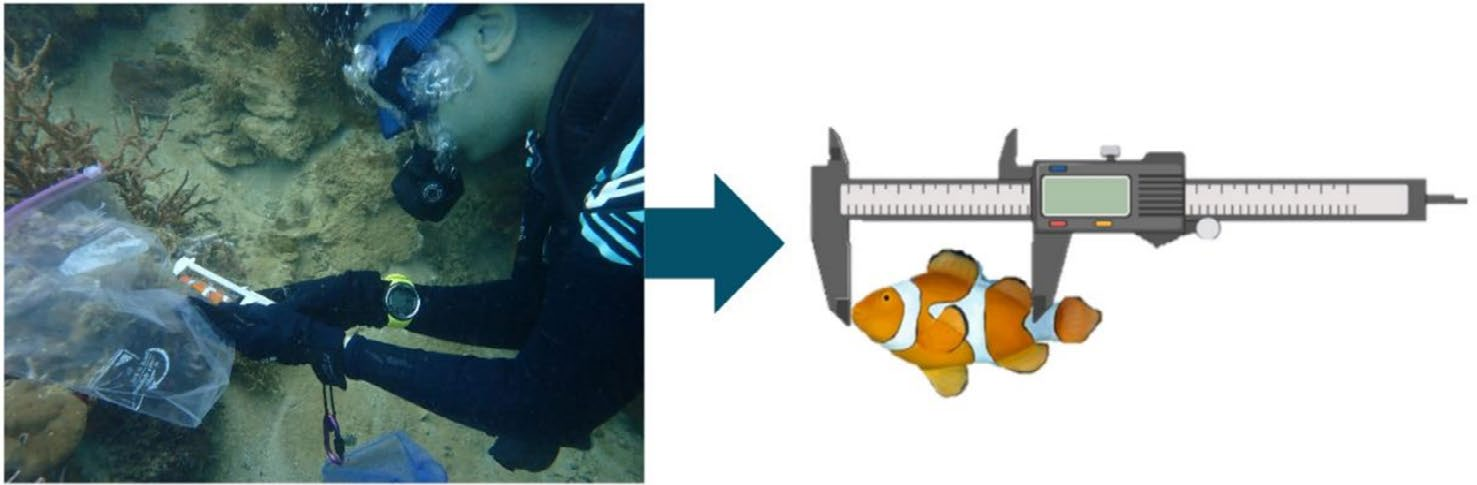
Photo and schematic depicting the method used to measure standard length (SL).

The set of cameras and acoustic loggers is non‐invasive and has very low impact on anemonefish and other surrounding species welfare and behaviour. After the recordings, all individuals of the group were caught with hand nets and measured underwater with callipers to obtain their standard length (SL) in mm (Figure 3).

### 2.3 Video and Audio Analysis

For the analysis, 12 min of continuous footage and audio from the raw recordings were selected, excluding sections where anthropogenic noise was present (such as diver or boat noise), as well as 5 min following any such disturbance. The video footage was reviewed in BORIS (v. 7.12) (Friard and Gamba 2016) to identify the individuals producing vocalisations and the behaviours associated with them, while Audacity (v. 3.7.3) (Azalia et al. 2022) was used to analyse the corresponding audio data and document the vocalisations. Both tools were used in combination to match each vocalisation to the individual that produced it. Video and audio data were synchronised using taps made by the divers, which were captured by both the camera's audio and the acoustic logger. A low‐pass filter with a threshold of 2 kHz was applied in Audacity to remove higher frequency noise and enhance the clarity of the vocalisations.

The behaviours scored were divided into categories (based on Wong et al. (2013)): (i) intraspecific aggression; (ii) submission; (iii) neutral displays; (iv) interspecific aggression; and (v) territorial displays. Intragroup aggression included aggressive interactions directed towards conspecifics within the social group. Submission included behaviours indicating yielding or avoidance in response to aggression or to appease higher ranked individuals. Neutral displays were defined as non‐agonistic encounters between individuals within one body length of each other, including swimming together, meeting, following or touching (Yllan et al. 2024). Interspecific aggression encompassed aggressive behaviours directed towards heterospecifics, including displays towards potential egg predators (e.g., Labridae), anemone predators (e.g., Chaetodontidae) or food competitors such as other Pomacentridae. Territorial displays were defined as vocal behaviours performed at the edge of the anemone, without direct interaction with other individuals.

Individuals were recognised by their natural markings (specifically, the unique patterns of their colour bands) which vary between individuals in this species, as well as by size differences between ranks due to the strict size hierarchy within groups. Visual recognition of individuals based on these traits is a widely used method in research on this genus (Laudet and Ravasi 2023). Vocalising individuals were confirmed by visible jaw movements at the time of sound production. Any vocalisations for which the producing individual could not be clearly identified, or for which no visible jaw movement was observed, were excluded from the analysis. Vocalisations were labelled in relation to the rank of the vocalising individual and the behaviour observed. Labelled acoustic data in format .*wav* was cut in smaller clips with Audacity and imported to Raven Pro v 1.6 (Charif et al. 2010) to extract the spectrogram parameters of all vocalisations, which were exported in selection tables.

### 2.4 Statistical Analysis

The statistical analyses were conducted using R version 4.4.2 (RStudio Team 2023). The *warbleR* and *Rraven* packages (Araya‐Salas 2020; Araya‐Salas and Smith‐Vidaurre 2017) were used to import the selection tables from Raven and to extract and quantify specific acoustic features. The audio recordings were sampled at 48 kHz in stereo with 16‐bit resolution, analysed using a Hann window, a 1024‐point Fast Fourier Transform (FFT), and a 50% window overlap. The *seewave* package (Sueur et al. 2008) was used to visualise the acoustic data, including the generation of spectrograms and oscillograms.

**FIGURE 4:**
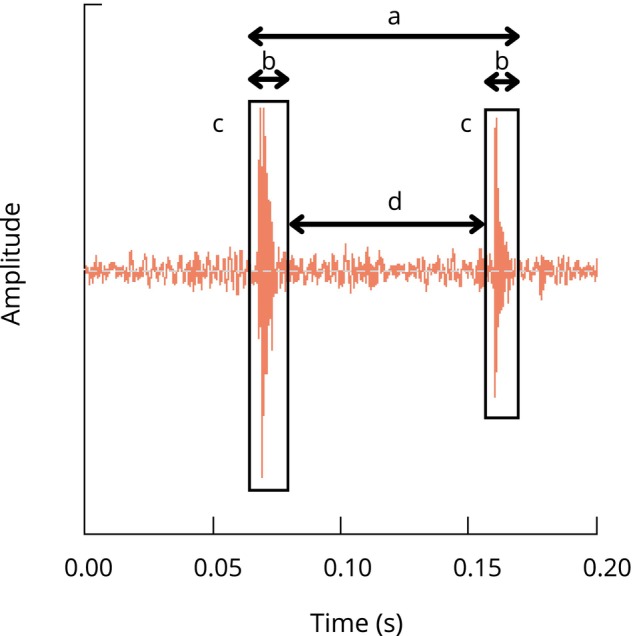
Oscillogram illustrating the temporal structure of a fish vocalisation. (a) indicates the vocalisation duration, measured from the start of the first pulse to the end of the last pulse. (b) Marks the pulse duration, representing the length of each individual acoustic pulse. (c) Denotes the pulses within the vocalisation. (d) Shows the pulse interval, defined as the time between the end of one pulse and the beginning of the next.

For the analysis the following acoustic parameters (Figure 4) were selected: (1) vocalisation duration (s), (2) pulse interval (s), (3) number of pulses, (4) pulse duration (s), (5), peak frequency (kHz), (6) dominant frequency (kHz) and (7) entropy. The temporal parameters were measured from oscillograms, while spectral and frequency measurements were derived from power spectra. Vocalisation duration was defined as the time from the first to the last pulse of a vocalisation. The number of pulses within each vocalisation was manually counted using oscillograms. Pulse duration was measured as the time between the start and end of each pulse, and pulse interval was defined as the time between the end of one pulse and the beginning of the next. Spectral parameters included entropy, a measure of the signal's spectral complexity or randomness; dominant frequency, the average frequency of the most prominent spectral peaks over time; and mean peak frequency, the average frequency of the highest energy peaks across the vocalisation. Vocalisation duration and number of pulses were measured per vocalisation, whereas all other parameters were measured per pulse or interval. Each vocalisation was given a unique ID to account for repeated measures within the same vocalisation train.

To compare how all acoustic parameters differ between behaviours, size and social rank we performed a Permutational Multivariate Analysis Of Variance (PERMANOVA), using the *adonis2* function of the *vegan* package (Oksanen et al. 2008). This method was selected due to violations of parametric assumptions such as multivariate normality and homogeneity of covariances, which were assessed via Shapiro–Wilk tests on residuals and Box's M test. We tested the homogeneity of multivariate dispersions to ensure that the assumptions for PERMANOVA were met.

We fitted Generalised Linear Mixed Models (GLMMs) using the *glmmTMB* (Brooks et al. 2017) package in R, to examine the effects of body size (SL) and behavioural category on acoustic parameters. We also fitted models to further explore the effect of body size (continuous predictor) and social status (categorical predictor with three levels: Female, Male, Subordinate), with SL centred to account for correlation between body size and social rank. Separate models were constructed for each response variable (vocalisation duration, number of pulses, pulse duration, pulse interval, peak frequency, dominant frequency and entropy). Group ID was included as a random effect for models of vocalisation duration and number of pulses, while both group ID and vocalisation ID were included as random effects for the remaining variables to account for non‐independence within vocalisation trains. The distribution of each response variable was assessed, and an appropriate family was selected: Gaussian for normally distributed data, negative binomial for count data, and Gamma for positive, continuous, non‐normally distributed data. Model selection was guided by Akaike's Information Criterion (AIC), and likelihood ratio tests were used when models differed by less than 2 AIC units (ΔAIC < 2). Pairwise comparisons between factor levels were performed using the *emmeans* package (Lenth 2023) with Tukey's method to adjust for multiple comparisons. Finally, model explanatory power was evaluated using the *performance* package (Lüdecke et al. 2021), which provided marginal R^2^ (variance explained by fixed effects) and conditional *R* ^2^ (variance explained by both fixed and random effects), following the approach of Nakagawa et al. (Nakagawa et al. 2017).

## 3 Results

### 3.1 Social Context of Vocalisations

**FIGURE 5:**
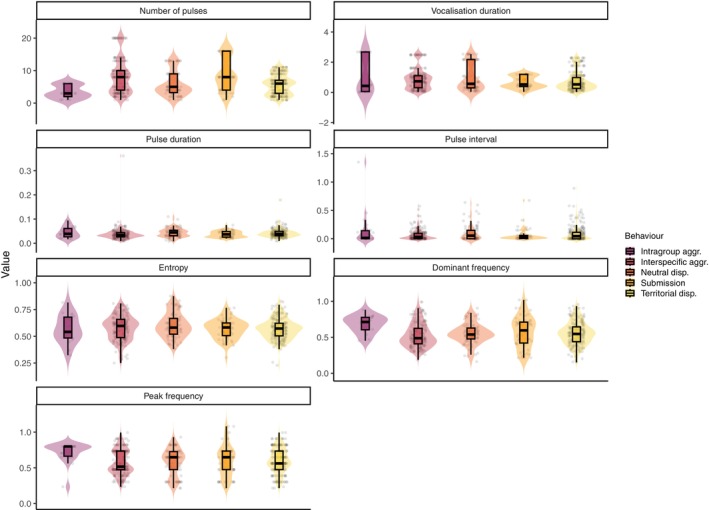
Distribution of acoustic parameters across behavioural categories. Violin plots show the full distribution of each acoustic parameter (number of pulses, vocalisation duration, pulse duration, pulse interval, entropy, dominant frequency and peak frequency) for five behavioural contexts: intragroup aggression, interspecific aggression, neutral displacement, submission and territorial display. Boxplots indicate medians and interquartile ranges, and points represent individual observations.

We identified five behaviours that were associated with vocalisations of *A. percula* : interspecific aggression (aggressive displays to other fish species), intragroup aggression (aggressive displays to other fish within the social group), neutral interactions (non‐agonistic interactions between members of the social group), submission (submissive displays to other members of the social group) and territorial displays, in which individuals would get close to the edge of the anemone and produce vocalisations without a direct interaction with another fish (see Table 1 and Figure 5).

**TABLE 1:** Table showing the mean (± standard deviation (sd)) of frequency, peak frequency, dominant frequency, entropy, duration, pause duration and pulse number of all the behaviours scored in *Amphiprion percula* .

Behavioural category	Vocalisation duration (s)	Entropy	Pulse interval (s)	Pulse duration (s)	Number of pulses	Peak frequency (kHz)	Dominant frequency (kHz)
	Mean ± SD	Mean ± SD	Mean ± SD	Mean ± SD	Mean ± SD	Mean ± SD	Mean ± SD
Intragroup aggression	1.050 ± 1.200	0.566 ± 0.130	0.160 ± 0.325	0.044 ± 0.023	3.440 ± 2.010	0.720 ± 0.143	0.684 ± 0.120
Interspecific aggression	0.872 ± 0.683	0.579 ± 0.115	0.066 ± 0.091	0.037 ± 0.026	8.160 ± 5.310	0.576 ± 0.172	0.526 ± 0.162
Neutral displays	0.965 ± 0.899	0.602 ± 0.114	0.102 ± 0.142	0.043 ± 0.018	5.850 ± 3.800	0.581 ± 0.185	0.550 ± 0.147
Submission	0.658 ± 0.389	0.568 ± 0.091	0.062 ± 0.106	0.037 ± 0.013	8.690 ± 5.030	0.598 ± 0.217	0.573 ± 0.215
Territorial displays	0.726 ± 0.622	0.570 ± 0.095	0.087 ± 0.126	0.041 ± 0.017	5.680 ± 3.000	0.599 ± 0.170	0.553 ± 0.157

Aggressive vocalisations directed towards other species (interspecific aggression) were characterised by the highest number of pulses (8.16 ± 5.31), but relatively short pulse durations (0.037 ± 0.026 s) and intervals (0.066 ± 0.091 s), suggesting rapid sequences of calls. In contrast, intragroup aggression showed fewer pulses per vocalisation (3.44 ± 2.01) but longer vocalisation durations (1.05 ± 1.20 s), indicating that these calls were longer and potentially more energetically costly.

**FIGURE 6:**
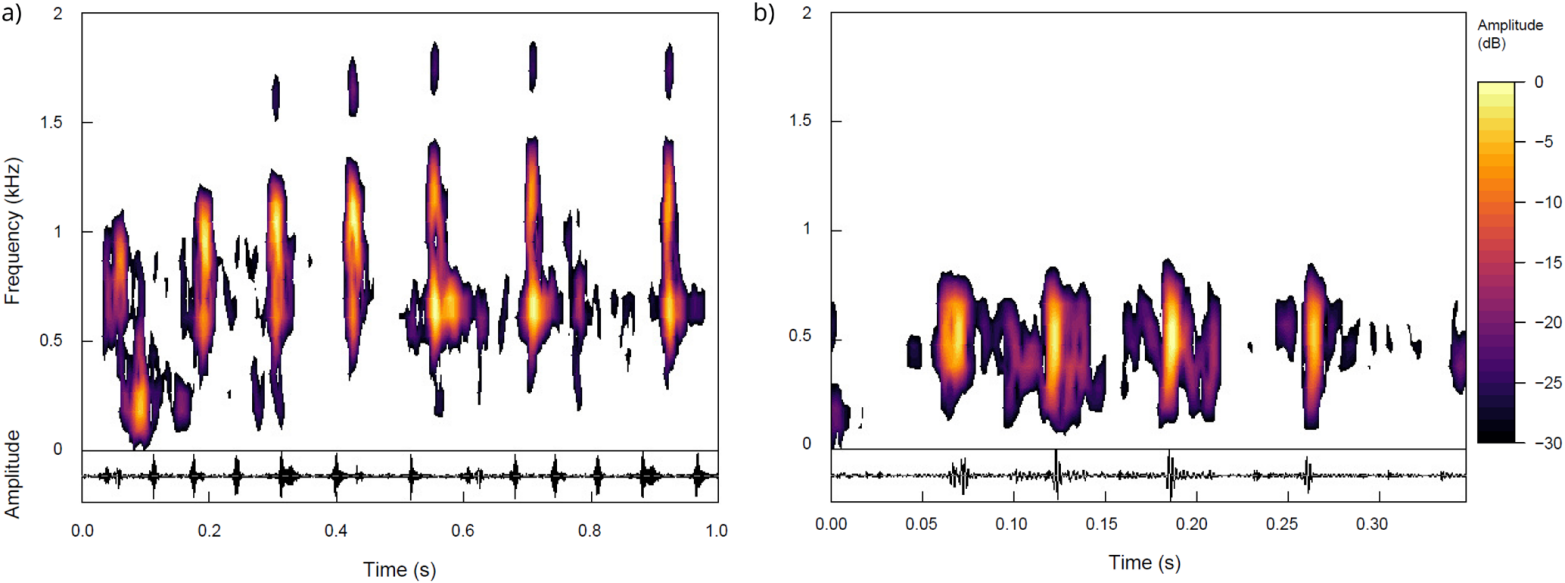
Spectrograms and oscillograms of *A. percula* vocalisation. The y‐axis represents frequency (in kHz) and amplitude, while the x‐axis represents time (in seconds). The spectrogram on the left (a) corresponds with a submissive vocalisation while the spectrogram on the right (b) corresponds with an aggressive vocalisation.

Neutral interactions had intermediate acoustic characteristics, with moderate pulse numbers (5.85 ± 3.80) and vocalisation durations (0.965 ± 0.899 s), while submission vocalisations were marked by a high number of pulses (8.69 ± 5.03) but relatively short pulse durations (0.037 ± 0.013 s) and intervals (0.062 ± 0.106 s), reflecting fast, possibly repeated submissive signals (Figure 6). Territorial displays were intermediate in almost all measured acoustic parameters, with moderate numbers of pulses (5.68 ± 3.00) and durations (0.726 ± 0.622 s).

Spectral characteristics also varied among behaviours. Aggression was associated with the highest peak (0.720 ± 0.143 kHz) and dominant frequencies (0.684 ± 0.120 kHz), whereas interspecific aggression showed the lowest frequencies (peak: 0.576 ± 0.172 kHz, dominant: 0.526 ± 0.162 kHz), suggesting that different behaviours may be differentiated by frequency as well as temporal structure. Entropy values were generally similar across behaviours (0.566–0.602), indicating comparable levels of complexity or noisiness in the calls.

### 3.2 Differences in Acoustic Profiles

The acoustic profiles of individuals varied significantly according to behavioural category, body size and social status. There was a significant effect of behaviour on multivariate acoustic structure (*F*_4,586_ = 4.87, *p* < 0.001, *R* ^2^ = 0.031), while size (*F*_1,586_ = 8.96, *p* < 0.001, *R* ^2^ = 0.014) and social status (*F*_2,586_ = 6.93, *p* < 0.001, *R* ^2^ = 0.022) also accounted for significant but relatively small proportions of variation. Although the majority of variance was residual (~93%), these results indicate consistent differences in acoustic structure related to behavioural context, body size and social hierarchy.

#### 3.2.1 Effect of Behavioural Context and Size on Acoustic Features

Body size (standard length, SL) and behavioural category significantly influenced the acoustic properties of *A. percula* vocalisations, with several features showing an interaction between SL and behaviour. The interaction between behavioural category and SL had a significant effect on pulse number, vocalisation duration and entropy (pulse number: $\chi_{4}^{2}$ = 12.56, *p* < 0.05, $R_{c}^{2}$ = 0.264, $R_{m}^{2}$ = 0.176; duration: $\chi_{4}^{2}$ = 21.42, *p* < 0.001, $R_{c}^{2}$ = 0.311, $R_{m}^{2}$ = 0.192; entropy: $\chi_{4}^{2}$ = 20.27, *p* < 0.001, $R_{c}^{2}$ = 0.439, $R_{\mathrm{cm}}^{2}$ = 0.102). Vocalisation duration, pulse number and entropy all increased with standard length overall (duration: 0.223 ± 0.065, *p* < 0.001; pulse number: 0.079 ± 0.044, *p* = 0.071; entropy: 0.025 ± 0.008, *p* < 0.01), but these positive effects of size were reduced in interspecific aggression, neutral and territorial contexts for all three traits, and additionally in submission contexts for entropy (all *p* < 0.05). Dominant frequency, peak frequency and pulse duration were not significantly affected by SL or behaviour. Pulse interval differed among behaviours ($\chi_{4}^{2}$ = 9.99, *p* < 0.05, $R_{m}^{2}$ = 0.068), with interspecific aggression and submission showing shorter intervals, but was not influenced by SL.

**FIGURE 7:**
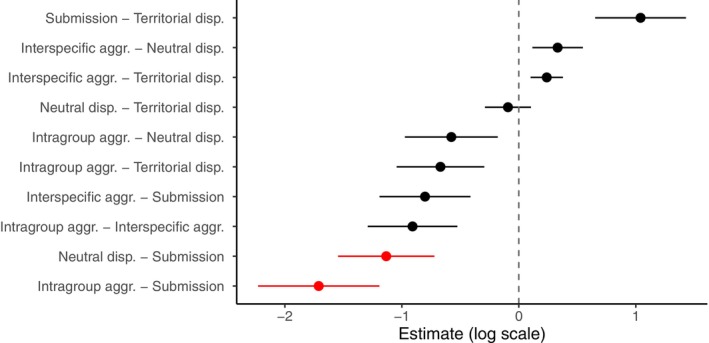
Pairwise comparisons of pulse number across behavioural categories in *A. percula* . Points represent estimated differences on the log scale, with error bars showing ±1 standard error. Red points indicate significant differences (*p* < 0.05) between categories, while black points indicate non‐significant differences.

The pulse number of vocalisations differed significantly across behavioural contexts (Figure 7). Submissive calls contained significantly more pulses than aggressive (estimate = −1.711 ± 0.519, *p* < 0.01) and neutral vocalisations (estimate = −1.134 ± 0.412, *p* < 0.05). A similar trend was observed when comparing submission to territorial calls (estimate = 1.041 ± 0.388, *p* = 0.057), though this difference was marginally non‐significant. No significant differences were detected between aggression, interspecific aggression, neutral and territorial contexts (all *p* > 0.1). These results indicate that submission is the main behavioural context in which pulse number is markedly elevated, distinguishing it from other vocal displays.

#### 3.2.2 Effect of Size and Social Status on Acoustic Features

Centred body size (SLc) and social status had limited effects on the acoustic properties of *A. percula*. Social rank had a significant effect on dominant frequency ($\chi_{2}^{2}$ = 6.96, *p* < 0.05, $R_{c}^{2}$ = 0.604, $R_{m}^{2}$ = 0.051), with males and subordinates tending to produce higher frequencies than females, while SLc showed a marginal positive effect (estimate = 0.009 ± 0.005, *p* = 0.086). Entropy decreased slightly with increasing SLc (estimate = −0.005 ± 0.002, *p* < 0.05, $R_{c}^{2}$ = 0.443, $R_{m}^{2}$ = 0.023), but was not influenced by social rank. Pulse duration showed a marginal trend with SLc ($\chi_{1}^{2}$ = 3.13, *p* = 0.077). There was no significant effect of SLc or social rank on pulse number, mean peak frequency, pulse duration or interval duration between pulses (all *p* > 0.05). No significant effect of the interaction between SLc and social rank was found.

**FIGURE 8:**
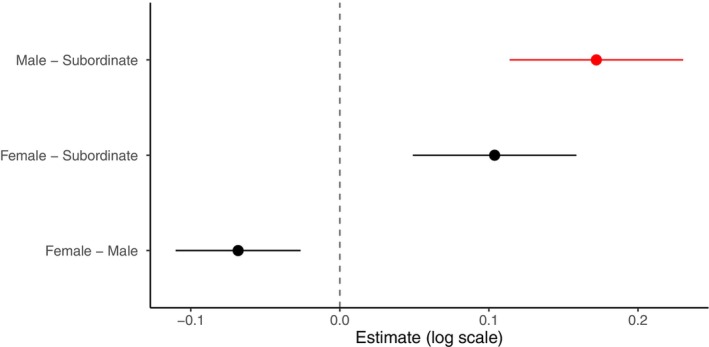
Pairwise comparisons of dominant frequency across social status in *A. percula* , controlling for centred body size (SLc). Points represent estimated differences on the log scale, with error bars showing ±1 standard error. Red points indicate significant differences (*p* < 0.05), while black points indicate non‐significant differences.

When comparing social ranks, subordinate individuals consistently differed from males, particularly in dominant frequency (Figure 8). Males tended to produce slightly higher frequencies than females, but this difference was not significant (estimate = −0.068, *p* = 0.23), and no significant differences were observed between subordinates and females (estimate = 0.104, *p* = 0.14). Subordinates produced significantly higher mean frequencies than males (estimate = 0.172, *p* < 0.01). These results suggest that social rank has a modest effect on dominant frequency, with the primary difference occurring between subordinate and male individuals, while females do not differ significantly from either group.

## 4 Discussion

Our study provides one of the first comprehensive in situ examinations of acoustic communication and associated behaviours in wild anemonefish ( *Amphiprion percula* ), revealing how vocalisations vary with behavioural context, body size and social rank. Our results highlight how acoustic signalling might play a key role in maintaining social structure and mediating interactions within natural groups.

Our observations showcase that anemonefish, in their natural habitat, use vocalisations frequently and in a wide range of contexts. Vocalisations, alongside visual displays, were used by group members to signal to each other, not only during agonistic encounters but also during neutral/social interactions. This is consistent with previous observations (Parmentier and Lecchini 2022; Colleye and Parmentier 2012), however, most studies have focused on agonistic sounds, and few have mentioned the presence of social sounds (Amorim 2006; Crawford et al. 1997; Banse et al. 2024). We have also observed anemonefish produce vocalisations when interacting with other species and producing vocalisations on the edge of the anemone without directly interacting with other fish, something that has not been described previously in anemonefish. Vocalisations had distinct acoustic differences among behaviours. These results are consistent with the hypothesis that acoustic signals convey social information and mediate hierarchical interactions (Reddon et al. 2021). Submissive calls were characterised by a higher number of pulses than aggressive, neutral or territorial vocalisations. Rapid, multi‐pulsed submissive vocalisations may function to signal yielding without escalating physical aggression, thereby reducing the risk of injury and maintaining group cohesion. In contrast, aggressive vocalisations, particularly intragroup aggression, were longer in duration but contained fewer pulses. Thus, the vocalisations of *A. percula* differed between behavioural contexts, highlighting the importance of acoustic communication as a mechanism for maintaining cohesion within social groups.

The acoustic profiles of *A. percula* varied consistently with behavioural context, body size and social status, indicating that vocalisations provide contextual and intrinsic information. Behavioural context was the strongest predictor of acoustic variation, suggesting that these signals are central for mediating interactions in a species where stable dominance hierarchies are essential to group functioning. Body size also influenced vocal features, in line with previous findings that found a correlation between size and acoustic features (Colleye et al. 2009, 2011). However, we additionally found a significant effect of social rank, raising the possibility that differences attributed solely to physical traits in earlier studies may also reflect hierarchical position. Because size and rank are tightly correlated in anemone societies (Buston and Cant 2006), disentangling their respective influences on call structure remains challenging. These results therefore point to the need for further study to determine whether vocal features primarily reflect morphological constraints, social dynamics or an interaction between the two and reinforce the idea that acoustic cues play a key role in regulating group cohesion in this highly social species.

Our analysis controlling for body size indicates that while morphology constrains vocal features, the expression of those features is strongly shaped by social context and the functional demands of each interaction. Although larger individuals generally produced longer calls with more pulses and higher entropy, these size‐related effects were reduced in certain contexts, including interspecific aggression, neutral, territorial, and, for entropy, submissive displays. Pulse interval varied only with behaviour, with interspecific aggression and submission characterised by shorter intervals and therefore faster call sequences. In contrast to previous studies (Colleye et al. 2009; Myrberg et al. 1993), frequency was not strongly influenced by body size, suggesting that acoustic scaling in anemonefish may be more context‐dependent than previously thought. These results suggest that vocalisations are not only reflections of morphology but are actively modulated by social context, challenging earlier assumptions about the strong correlation of size and reinforcing the idea that acoustic cues play an important role in mediating behaviour and maintaining group cohesion in this species.

Also, comparisons across social ranks revealed that subordinate individuals consistently differed from males in their vocal profiles, particularly in dominant frequency. Subordinates produced significantly higher mean frequencies than males, while males tended to produce slightly higher frequencies than females, though this difference was not significant. Interestingly, the difference between females and subordinates was similar in magnitude to the male–subordinate difference but did not reach statistical significance. This pattern underscores that vocalisations may convey information about hierarchical position, particularly in distinguishing subordinates from dominant males, even when differences involving females are more subtle.

The number of pulses per vocalisation varied significantly across behavioural contexts, with submission standing out as the only context in which pulse number was significantly higher. This is consistent with the classification of anemonefish sounds made by Colleye and Parmentier (2012). Since anemonefish produce vocalisations using the jaw teeth, a mechanism which does not offer great flexibility unlike other taxa sound‐producing mechanisms, we suggest that variation in pulse number might provide an adaptable way to create acoustic differences between calls. Producing a higher number of pulses may thus serve as a distinctive submissive signal, providing a clear cue to dominant individuals and reducing the likelihood of costly escalation within the social group (Reddon et al. 2021). Changes in pulse interval might also help create variation in calls (Colleye et al. 2011). Although we found a significant effect of behavioural category in pulse interval, pairwise comparisons did not reveal significant differences in pulse interval between behaviour pairs.

Our study demonstrates that acoustic communication in *A. percula* is highly context‐dependent, shaped by behaviour, body size, and social rank, and plays a key role in maintaining social hierarchies and group cohesion. By conducting field‐based observations in natural habitats, we were able to link vocalisations directly to behaviour and social structure, capturing ecological and social complexities that laboratory studies alone cannot replicate, including habitat acoustics, social interactions and environmental noise. Future research should focus on experimentally testing the functional significance of these vocalisations through playback experiments and examine how environmental factors such as noise pollution influence vocal behaviour. Such investigations will deepen our understanding of the adaptive role of acoustic communication in fishes and provide critical insights for behavioural ecology and conservation in increasingly human‐impacted coral reef ecosystems.

## Author Contributions

**Lucia Yllan:** conceptualization (lead), data curation (lead), formal analysis (lead), investigation (lead), methodology (lead), visualization (lead), writing – original draft (lead), writing – review and editing (equal). **Theresa Rueger:** conceptualization (supporting), formal analysis (supporting), funding acquisition (lead), supervision (lead), writing – review and editing (equal).

## Ethics Statement

Ethical clearance was given by Newcastle University Ethics Committee.

## Conflicts of Interest

The authors declare no conflicts of interest.

## Data Availability

The data supporting the findings of this study are openly available in *Mendeley Data* at https://doi.org/10.17632/7m9v6hxzrr.1, under the title ‘Amphiprion percula vocalizations data’ (Yllan et al. 2024).
